# Determinants of community health fund membership in Tanzania: a mixed methods analysis

**DOI:** 10.1186/s12913-014-0538-9

**Published:** 2014-11-20

**Authors:** Jane Macha, August Kuwawenaruwa, Suzan Makawia, Gemini Mtei, Josephine Borghi

**Affiliations:** Ifakara Health Institute, P.O BOX 78373, Plot 463, Kiko Ave., Mikocheni, Dar es Salaam, Tanzania; London School of Hygiene and Tropical Medicine, 15-17 Tavistock Place, London, WC1H 9SH UK

**Keywords:** Community based health insurance, Community health fund, Enrolment, Adverse selection, Sustainability, Quality of health services

## Abstract

**Background:**

In many developing countries, initiatives are underway to strengthen voluntary community based health insurance as a means of expanding access to affordable care among the informal sector. However, increasing coverage with voluntary health insurance in low income settings can prove challenging. There are limited studies on determinants of enrolling in these schemes using mixed methods. This study aims to shed light on the characteristics of those joining a community health fund, a type of community based health insurance, in Tanzania and the reasons for their membership and subsequent drop out using mixed methods.

**Methods:**

A cross sectional survey of households in four rural districts was conducted in 2008, covering a total of 1,225 (524 members of CHF and 701 non-insured) households and 7,959 individuals. In addition, 12 focus group discussions were carried out with CHF members, non-scheme members and members of health facility governing committees in two rural districts. Logistic regression was used to assess the determinants of CHF membership while thematic analysis was done to analyse qualitative data.

**Results:**

The quantitative analysis revealed that the three middle income quintiles were more likely to enrol in the CHF than the poorest and the richest. CHF member households were more likely to be large, and headed by a male than uninsured households from the same areas. The qualitative data supported the finding that the poor rather than the poorest were more likely to join as were large families and of greater risk of illness, with disabilities or persons with chronic diseases. Households with elderly members or children under-five years were also more likely to enrol. Poor understanding of risk pooling deterred people from joining the scheme and was the main reason for not renewing membership. On the supply side, poor quality of public care services, the limited benefit package and a lack of provider choice were the main factors for low enrolment.

**Conclusions:**

Determinants of CHF membership are diverse and improving the quality of health services and expanding the benefit package should be prioritised to expand voluntary health insurance coverage.

## Background

In recent years, community based health insurance has been promoted within health financing reforms in many developing countries [1,2] as a mechanism for raising additional funds for essential public health services, enhancing access to care and reducing out-of-pocket payments [1,3-5]. Community based health insurance is usually organised and managed by local government institutions, local health facilities, or non-government organisations, with significant community involvement [1,3,6]. Enrolment in such schemes is voluntary, and premiums tend not to be according to ability to pay, nor are they risk rated, with schemes often running on a non-profit basis [3]. Revenue collection mechanisms vary from place to place. In some cases, contributions are collected at a specific period in the year, such as during the harvest season [7], in other cases people can join year round [4]. Schemes sometimes promote individual enrolment, and in other cases couples and their children are enrolled together [8].

Despite the proven affects of community based health insurance schemes in enhancing access to services and financial protection [3,9,10], in many developing countries there has been limited population enrolment in such schemes [2-5]. Indeed, in Sub-Saharan Africa coverage rarely attains more than 10% of the target population [5] with some exceptions in countries such as Rwanda and Ghana [11-14].

A number of studies have examined and reviewed the constraints to increasing enrolment [3-5,8,9,14-18]. Among the potential barriers to enrolment are affordability of premiums [15,19], poor quality of health care services offered to members and limited referral services [4]. Poor management (unfavourable scheme design) and lack of trust were also reported in some studies to affect the coverage of community based insurance schemes [15,20,21].

Previous studies have employed quantitative methods such as household surveys [2,5,16,17] or qualitative methods, such as focus group discussions [18,22] to examine factors determining enrolment and barriers. Household surveys can be valuable in exploring household characteristics associated with insurance membership, but are less able to shed light on why these patterns are observed, nor are they able to readily unpack the supply side and institutional barriers to enrolment; which are better examined through qualitative methods. There is limited evidence on the determinants of community insurance membership using mixed methods enabling a parallel assessment of demand and supply side factors underlying enrolment. To address this gap, in this study we adopted a mixed methods approach to enable an in-depth examination of membership determinants and demand and supply side factors explaining enrolment and drop out of the Community Health Fund (CHF), a community based health insurance scheme in Tanzania after more than 10 years of implementation. A previous study was carried out in Tanzania, which examined the supply side/institutional barriers to enrolment, through an assessment of implementation at different levels of the system [4], however, there has been no subsequent analysis of these factors, nor an assessment of the characteristics of insured members in this context.

## Methods

### Study setting

Tanzania introduced the Community Health Fund (CHF), a form of voluntary community based health insurance for the rural informal sector in 2001 [4]. The household is the unit of enrolment and the majority of districts have set their contribution rate to between US$ 4.2 and US$ 12.7^a^ per year [23]. The premium rate is the same for all CHF members within a district but varies across districts. CHF members register at public health facilities where premiums are also collected and member households are subsequently eligible for free primary care at the selected facility. In some districts limited referral health care services are also covered. After collection, the funds are deposited into a cost sharing or CHF account at the council level [24], and in some districts a percentage of the funds goes back to the facility and can be used for drug purchases and minor repairs. To supplement the revenue generated through household contributions, the government matches all contributions by members through a matching grant. Some of the conditions for accessing the matching grant include having membership records, by-laws and functioning CHF institutional arrangements [25]. Matching grants are allocated based on the amount of premium revenue each district collects, thus those less able to generate contributions receive less funds [26].

A system of exemptions aimed at ensuring free access to public health services among vulnerable groups such as children under-five years of age, pregnant women, and people with chronic diseases like HIV/AIDS also exists. A waiver system is in place to protect those who are unable to pay user fees and to join the CHF, although in practice this is poorly implemented [27-29].

Council Health Service Boards (CHSBs) and health facility governing committees (HFGCs) operate at the district level and at the facility level respectively. Their role is to improve accountability between health care providers and communities, ensuring exemptions are respected and mobilising resources from communities, such as CHF premiums and administering them. To date, national CHF coverage remains very low at about 7.1% of the total population [30], compared to the population of informal sector workers and their dependents, which represent more than 70% of the entire population. However, the government is committed to extending coverage through the CHF [25].

### Data collection and analysis methods

#### Quantitative data

A cross sectional household survey was conducted in 4 Districts Councils (Singida, Mbulu, Kigoma rural and Kilosa) in 2008, covering a total of 1,225 (524 members of CHF and 701 non-insured) households and 7,959 individuals. The districts were selected purposively such that they had at least 10% of their population covered by the CHF. Two districts (Kigoma and Kilosa) offered the basic benefit package to members (outpatient care, mainly limited to primary facilities), and two (Singida and Mbulu) had expanded benefits which included inpatient care.

A stratified random sample of facilites was selected within each district based on distance from the district headquarters for identification of CHF members. We selected two nearby facilities (less than 30 minutes by car), two medium distance (between 30 minutes and one hour) and one far away (more than an hour). We compiled a list of all CHF member households that had been a member for at least a year from records at the sampled health facilities. We then took a random sample of 30 households from the list. Village leaders assisted in the identification of the sampled households in the community. Village leaders also provided a list of all the non-insured households living within the same hamlets as the identified CHF members. From this list, 35 non-insured households were picked at random.

The household survey compiled information on household demographic and social economic characteristics among other variables. Descriptive analysis of the characteristics of CHF members and the uninsured was first done, tests for differences in means and proportions were done using Adjusted Wald F-test. A marginal effects logit model was run to identify the household characteristics associated with CHF membership. The reporting of standard errors adjusts for clustering at the facility level. We ran a series of diagnostic tests to ensure the model was correctly specified, to check for goodness of fit, multicollinearity and heteroskedasticity. The analysis was done using STATA 11 software.

### Qualitative data

Qualitative data were collected to examine in more depth the demand side constraints to enrolment, as well as to explore the extent of supply side factors affecting enrolment and drop-outs. Qualitative data were collected in two districts: Mbulu and Kigoma rural. Among those dispensaries selected to collect household survey data, two facilities were selected; one that was close and one that was faraway to collect qualitative data. At the dispensaries, 7 to 10 CHF members’ cards were selected at random and members invited to a focus group discussion (FGD). Village leaders helped to randomly locate non members from the same area. Focus group discussions were also conducted with HFGC members. A total of 12 FGDs were carried out with CHF members, non-scheme members and members of the HFGC (Table 1). FGDs were conducted in Kiswahili and tape recorded with written consent from participants. The data were then transcribed and translated into English.

**Table 1:** **Overview of FGD’s carried out by district and stakeholder type**

**Stakeholder type**	**District**		**Total**
	**Mbulu**	**Kigoma**	
Health facility committee members	2	2	4
CHF members	2	2	4
Non-insured individuals	2	2	4
Total	6	6	12

Translated data were imported into QSR NVIVO 8 for coding and processing. Thematic content analysis was done, which entailed coding data according to key themes arising from the data. Initial coding was done by the lead author and a draft code book shared with other members of the team for comments. The code book had a range of themes, for example: characteristics of members of the CHF, reasons for joining the CHF, reasons for dropping out, and the ways adverse selection affects the scheme’s sustainability. Validation of the findings was done by triangulating and synthesizing data across respondent groups. Verbatim key quotations have been incorporated in the paper.

### Ethics and quality control

All data collection tools were pre-tested for quality control and field interviewers and supervisors were trained and supervised by two research coordinators. Debriefing meetings with survey interviewers were held at the end of each day to identify challenges and key issues arising from the field work. Ethical approval was obtained from the National Institute for Medical Research in Tanzania. The study was also approved by the Institutional Review Board of the Ifakara Health Institute. All respondents were given an information sheet in Swahili explaining the right to voluntary participation in the study and were asked to sign a consent form.

## Results

### Quantitative analysis results

#### Descriptive statistics

CHF member household heads were more likely than the heads of uninsured households to be male (89% versus 79%, p < 0.05); married (73% vs 59%, p < 0.05); older (46.4 versus 42.9 years, p < 0.1), completed secondary education and above (5% vs 3%, p < 0.1), be Christian (63% vs 55%, p < 0.001) and exempted (17% vs 14%, p < 0.001)(Table 2). CHF households were also more likely to have a higher number of members (7 vs 6, p < 0.001). There was also a higher proportion of CHF members than uninsured households in the sample from Mbulu district (28% versus 24%, p < 0.05) and Kigoma Rural (28% vs 25%, p < 0.001). There was no significant difference between CHF member households and the uninsured with respect to any of the other variables considered.

**Table 2:** **Characteristics of CHF members and non-member households**

**Variable**	**Variable description**	**Total N = 1,225**		**CHF member**		**Un-insured**	
				**n = 524**		**n = 701**	
		**N**	**%**	**n**	**%**	**n**	**%**
Gender of the household head,**	1 = male,	1,025	83	468	89	557	79
	0 = female						
Marital status of the household head,**	1 = married	796	65	383	73	413	59
	0 = otherwise						
**Wealth groups**							
Poorest, (reference group)	1 = Poorest Household	304	25	107	21	197	29
	0 = Otherwise						
Second poorest,		294	25	129	25	165	24
Middle,		275	23	122	24	153	22
Second richest,		222	19	102	20	120	17
Least Poor,	1 = Least Poor Household	102	9	49	10	53	8
	0 = Otherwise						
**Educational level of household head**							
Completed primary level education,	1 = Has completed primary school	921	75	397	78	542	77
	0 = Otherwise						
Secondary level education and above,*	1 = Has completed secondary education and above	49	5	27	5	22	3
	0 = Otherwise						
No formal education, (reference group)	1 = Has no formal education	189	15	75	14	114	16
	0 = Otherwise						
**Districts**							
Kigoma Rural,***	1 = Household comes from Kigoma rural district	325	27	149	28	176	25
	0 = Otherwise						
Kilosa	1 = Household comes from Kilosa district	246	20	71	13	175	24
	0 = Otherwise						
Mbulu,**	1 = Household comes from Mbulu district	323	26	149	28	1744	24
	0 = Otherwise						
Singida Rural	1 = Household comes from Singida Rural district	331	27	155	30	176	25
	0 = Otherwise						
**Employment status of household head**							
Employed in the formal sector,**	1 = Household head is employed in formal sector	47	4	28	5	19	3
	0 = otherwise						
Employed in the informal sector	1 = Household head is employed in the informal sector	1118	91	478	91	640	91
	0 = otherwise						
Not employed, (reference group)	1 = Household head is not employed	20	2	6	1	14	2
	0 = otherwise						
**Health status of the household head**							
Health poor	1 = Household head has poor self assessed health	114	9	53	10	61	9
	0 = otherwise						
Health average	1 = Household head has average self assessed health	361	30	175	34	186	27
	0 = otherwise						
Health Good, (reference group)	1 = Household head has good self assessed health	732	60	291	56	441	64
	0 = otherwise						
Religion of household head***	1 = Christian	718	58	330	63	388	55
	0 = Muslim, or Hindu/Buddhist or no religion						
Exemption eligibility of household head***	1 = eligible for exemptions	190	16	87	17	103	14
	0 = not eligible for exemptions						
**Continuous variables**							
Mean age of the household head in years, Mean [sd]*		1225	44.4 [13.3]	524	46.4 [12.7]	701	42.9 [13.5]
Mean number of children under 18, Mean [sd]		1225	3.6 [1.9]	524	4.0 [2.1]	701	3.4 [1.2]
Household size, Mean [sd]***		1225	6.2 [2.6 ]	524	7.0 [2.5]	701	5.5 [2.6]

*Significant at 10%; **Significant at 5%; ***Significant at 1%.

#### Determinants of CHF membership

When controlling for a range of variables that could explain the decision to enrol, wealth was found to be an important determinant (Table 3). Each wealth group was between 1-12% more likely to enrol in the CHF relative to the poorest group. However, the least poor were no more likely to join than the poorest. Larger households were more likely to be CHF members, with each additional household member increasing the chance of being a member by 4.3%. Christian’s were 6.7% more likely to be members than those from other religious groups. Test statistics showed that the model was correctly specified, and there was no evidence of heteroskedasticity or multicollinearity.

**Table 3:** **Logit model on the determinants of people joining CHF**

**Variable**	**Marginal effects coeff**	**p-values**
Gender*	0.109	0.059
Second poorest**	0.110	0.015
Middle*	0.096	0.091
Second richest	0.112	0.115
Least Poor	0.057	0.604
Age	0.010	0.273
Age squared	−0.000	0.565
Completed primary education	−0.002	0.950
Completed secondary education and above	−0.016	0.871
Household size***	0.043	0.000
Marital status	0.044	0.253
Religion*	0.067	0.096
Household head eligible for exemptions	−0.025	0.675
Number of observations	1,112	
Wald	155.46	
Prob	0.000	
Pseudo	0.078	
Log pseudolikelihood	−698.46	

*Significant at 10%; **Significant at 5%; ***Significant at 1%.

### Qualitative analysis results

#### Demand side factors explaining membership

The qualitative analysis revealed that household income, occupation, household size, poor health status of household members, and level of understanding of the concept of risk pooling were prominent demand side factors associated with enrolment.

During FGDs with all respondent groups there were mixed feelings as to whether the premium was fair and affordable for everyone in the community. For some, household income, and type of occupation, were said to be a positive predictor of joining the CHF, confirming the quantitative findings. People doing small business, farming and/or those having cattle were seen to be better able to pay the premium, compared to people without.

*“You can sell one or two hens and manage to pay 5000 Tanzanian shilling (Tsh), it’s cheap but not everybody has a business or cattle, thus [joining] becomes difficult” (FGD, Mbulu DC, HFGC members).*

In both districts it was observed that members of the National Health Insurance Fund for the formal sector^b^, which provides cover for civil servants, also enrol in the CHF to provide cover to their uninsured dependents.

*“We have teachers who are civil servants covered by the National Health Insurance Fund (NHIF) and they are also members of the CHF to extend coverage to other dependants” (FGD, Mbulu DC, HFGC members).*

Some FGD participants reported that the poor, those with low income and food insecurity would have difficulty affording the premium, especially during poor harvest seasons.

*“Some people are poor, so [they] can’t afford the premium and nowadays earnings from farming are very low” (FGD, Mbulu DC, HFGC members).*

*“We have many people who can’t even afford to buy food; it is not possible for them to pay Tsh 5000” (FGD, Kigoma DC, CHF members).*

However, there was a common view that the premium was less than the sum of out-of-pocket payments one would otherwise pay for more than one visit, which served as an incentive to join the scheme irrespective of income.

“*Everybody in this area is capable of paying this amount, Tsh 5000/=. We join the scheme because the premium is very low” (FGD, Kigoma DC, CHF member).*

*“User fees are expensive when you go to the dispensary several times in a year, compared to the CHF premium” (FGD, Mbulu DC, Uninsured).*

During the focus group discussions, participants also indicated that large households were more likely to join the CHF. Repeated illness or multiple concurrent illnesses within the household were additional perceived risk factors for joining the CHF, particularly among larger households.

*“The main reason for joining is having an extended family. If you join you start to experience a relief from those expenses, especially when you have to take care of more than one person, or repeated illness cases” (FGD, Kigoma DC, CHF members).*

*“One day I brought my son to the clinic, and a few days later my wife was also sick, though she was only given aspirin at the facility as there were no other drugs. She was supposed to go and buy outside the facility [other drugs prescribed by the doctor] but we didn’t pay at the facility because of the CHF card” (FGD, Mbulu DC, HFGC members).*

Large households with unstable income were reported to be likely to join the scheme to ensure access to health care when needed.

*“Due to the instability of my income, which depends on farming which is seasonal and unpredictable, I have no other choice; my whole family is looking at me, depending on me,” (FGD, Kigoma DC, HFGC members).*

Whilst health status did not emerge as a significant predictor of membership in the quantitative analysis, the qualitative data indicated that households with people with disabilities, older members, and with members suffering from chronic diseases such as epilepsy were joining the scheme more than other groups. Having vulnerable people in the family was seen as a push factor; these groups were seen to be more prone to multiple concurrent cases of illness or repeated illness, making membership more attractive.

*“My parents depend on me to take care of them when they fall sick, and I have my nephew with skin problems and my own children. Having a CHF card has been helpful especially to my nephew who visits the hospital most often” (FGD, Mbulu DC, HFGC members).*

“*If your dependants also experience severe illness often you will suffer even more. You will be paying money every day to buy drugs. That’s why people decide to join insurance though it doesn’t cover all you need [the benefit package is limited]” (FGD, Kigoma DC, CHF members).*

Poor understanding of risk pooling was reported to deter enrolment and membership renewal. For instance members felt that their contributions should be used to cover their own use of services, with the money remaining available to them if they did not seek care.

*“Why should I pay again to join for the next year, while I know my dependants didn’t fall sick this year? I didn’t use my money!” (FGD, Kigoma DC, Uninsured).*

### Supply side factors affecting membership

The main supply side factors identified to limit enrolment include quality of health services; access to the government matching grants, portability of the CHF membership card, limited access to referral services, and the exemption and waiver policy.

Low quality of health care at public facilities was one of the main factors discouraging people from joining or from renewing their insurance membership. The key aspects of quality reported by respondents were: the shortage of drugs, lack of diagnostic equipment, long waiting hours. When drugs are out of stock, CHF members have to buy drugs at private pharmacies.

*“Now, what made me drop out of this scheme it’s the shortage of drugs at the facility. And at the drug shop you can pay more than half of the fees that you paid to become a member of the scheme. This is double payment, it is better that I don’t join any more” (FGD, Mbulu DC, uninsured).*

*“When you go to the facility to be told to go and buy drugs at the private pharmacy, there is no difference between those who are insured and those un-insured” (FGD, Kigoma DC, CHF members).*

Many dispensaries have no diagnostic equipment. This resulted in bypassing to higher level referral facilities or private facilities that are not covered by the CHF.

*“Sometimes they don’t treat what was supposed to be treated at the dispensary, because there is no diagnostic equipment” (FGD, Mbulu DC, Uninsured).*

Uncertainty about the availability of medical supplies and limited working hours at the dispensary was also reported to affect membership levels.

*“....But there is no guarantee of the service provided. Today you get a complete service tomorrow half of the service, this is what discourages us” (FGD, Mbulu DC, CHF members).*

Another concern with public facilities was long waiting hours, which was thought to particularly affect CHF members, as they can only benefit from services at one health facility.

*“Often one can spend almost the whole day waiting at the facility, with only two staff to take care of everyone, it is not easy. CHF member can’t opt to go elsewhere, as they are restricted to one facility [where they first sign up to the scheme]” (FGD, Kigoma DC, Uninsured).*

In principle, the CHF premiums together with the government sponsored matching grant, was intended for districts to buy drugs and improve facility supplies. However, facilities reported difficulty accessing these supplies which combined with delays in the district getting the matching grant affected the efforts to improve services.

*“There is no improvement in the service, even if the government also contributes to the fund” (FGD, Kigoma DC, HFGC members).*

*“We were told if we contribute, the government would match our contribution by the same amount” (FGD, Mbulu DC, HFGC members).*

The limited benefit package was also reported to affect membership, both in terms of the lack of referral benefits (in districts where this was not covered) combined with the fact that cover is only provided at a single facility. This factor was particularly found to discourage the better off from joining as they could afford to access care at private facilities by paying out of pocket. It was indicated by most of the respondents that the referral services not covered by the CHF are more expensive than primary care services covered by the scheme.

*“The scheme only covers services at one facility, when you travel to other villages you will have to pay, and wait a long time to get attended to and that is discouraging” (FGD, Kigoma DC, uninsured).*

In contrast, the inclusion of referral services in the benefit package in Mbulu had motivated many people to join the scheme; particularly those living close to the referral facility, but the transport costs still discourages some members from going.

*“Hospital care is included in the CHF and the CHMT is working hard to ensure there are enough drugs for members, however you won’t always get the drugs, to be honest there are still some challenges” (FGD, Mbulu DC, HFGC members).*

*“Yes we have been told, we can go to the district hospital but the transport cost discourages members to go there” (FGD, Mbulu DC, CHF members).*

There were mixed views about the government’s exemption policy and its interaction with the CHF. Some saw the policy as deterring people from joining the CHF as public primary care is officially free for many services. However, there were also complaints that exemptions were often not fully implemented, and so it was felt by others that households would still join the scheme to increase their access to care.

*“There are a lot of free services […] sometimes people see no need [to join] because there are groups accessing free services” (FGD, Kigoma DC, CHF members).*

*“Sometimes it is better to join, as for instance old people are supposed to get free services, but when they visit the health facility they often pay. They provide only free childhood vaccinations and clinic services” (FGD, Mbulu DC, Uninsured).*

## Discussion

This study identified the demand and supply side factors affecting the decision to enrol and remain enrolled in the CHF in Tanzania. On the demand side, although the scheme is aimed at the informal sector, households who are employed in the formal sector were more likely to be members of the CHF. A study in Laos also found that married and employed household heads were more likely to be insured and remain insured [21]. Our study found an association between membership and the gender of the household head, contrary to a recent review by Acharya [9]. However, a study in Burkina Faso found that there was no effect of gender on enrolment [5] but that male headed households had a higher willingness to pay for insurance and female headed households were more likely to drop out [31,32].

Middle income groups were also more likely to join, with the poorest quintile, defined as having food insecurity, and least poor being less likely to join. The lack of benefit from community based health insurance to the poorest was also reported elsewhere [4,21]. Introducing a mechanism for exemption from premiums for those unable to pay could be used as an approach for reaching out to the poorest, and has been employed elsewhere [14]. In Tanzania, district councils are supposed to budget for the poor and provide free CHF cards to poor households, but in practice this does not occur due to the lack of clearly defined criteria for identifying the poor and a lack of earmarked funds to cover their costs. In order to promote such an approach, funds need to be made available to districts and the poor identified with support of community development personnel [28]. However, there are recognised challenges in effectively targeting the poor in low income settings in general and in Tanzania specifically [28,33].

The rich were more likely to opt out to have freedom to purchase services from private providers. This in some ways echoes the findings from a study in Ghana [14] that found the rich were more likely to enrol but also more likely to drop out due to dissatisfaction with care, and because they could afford alternative providers. These findings are nuanced compared to that reported in the review by Acharya and a recent study in Laos where income was found to be a positive predictor of enrolment [9,21]. Unlike other studies, education did not emerge as a key determinant of enrolment in the Tanzanian setting [9,14,21,31,32].

Our study highlighted evidence of adverse selection. Large households (especially those with children and/or elderly members); and people with chronic illness were more likely to join the scheme due to the greater perceived risk of care seeking. Risk of illness and risk of incurring health care costs were important factors underpinning the decision to join the CHF. Studies in China, Laos and Nicaragua also found evidence of adverse selection with sicker households being more likely to enrol [21,34,35]. In Burkina, households with few children were more likely to drop out of insurance [31], and in Senegal households with children under five years were more likely to enrol [36].

The findings indicate that the decision to join the CHF is typically based on a rational comparison of the premium and expected cost of care when paying out of pocket. Expected costs were higher for large households, or households with greater health risk (young children, elderly members, chronic conditions), explaining the greater likelihood of enrolling among these groups, with the exception of the very poorest who could not afford the premium and wealthier groups who prefer to maintain provider choice. The greater likelihood of households with sick individuals joining the CHF was not identified through the quantitative analysis, possibly due to the lack of sensitivity of our health status measure. The voluntary nature of community based health insurance [15] means it can be prone to adverse selection. Adverse selection is potentially concerning for the financial sustainability of community based health insurance, as those households within the risk pool have a greater than average expected treatment cost. Adverse selection also limits risk sharing between the wealthy and the poor [15]. While household rather than individual level enrolment has been reported to reduce adverse selection [5], this study has shown that adverse selection can also happen when enrolment is at the household level.

The design of community based health insurance (CBHI) can help address this issue. Currently, in many districts, the CHF has no mechanism to limit adverse selection. Households can join at any time, so can opt into the scheme when they become sick and need care. One district in Tanzania has established a waiting time (or a qualifying period) before which members can use health services [24], this has been reported to be an effective mechanism to contain the effect of adverse selection in CBHI [15]. The use of individual rather than household premiums would also be beneficial, as larger households are currently more likely to join. Another approach to avoid adverse selection is group enrolment, whereby groups such as co-operative members, or employers, or schools enrol together. This model operates in Uganda to cover students [37] and in some parts of Tanzania this approach has been highly effective in reducing drop outs compared to individual enrolment [24,38] and is effective in reducing adverse selection [39].

Finally, the findings indicated that lack of understanding of the risk pooling principle was a factor limiting enrolment and explaining drop out. Indications of limited understanding of the insurance concept, which includes risk pooling were also present in a study in Laos and Bukina Faso [21,40] and was reported to affect enrolment in a community health financing scheme in Uganda [41]. To address this issue further community sensitisation to raise awareness and understanding about the insurance concept is required. In Tanzania, there have been sensitization activities conducted by the National Health Insurance Fund (NHIF) who are currently managing the CHF to raise awareness among the population about the importance of pre-payments, which appears to have had had initial positive effects on enrolment [25].

Our study also identified a number of supply side factors constraining enrolment including a lack of drugs and diagnostic equipment at primary level facilities limited provider choice and a lack of referral care in some areas. Drug stock outs and lack of referral care results in incomplete financial protection for households. To address the problem of drug stock outs some districts have introduced buffer stocks, whereby the district buys additional drugs, often using CHF revenue. Facilities are also allowed to access reserved drugs at the district headquarters during stock outs in addition to the regular drug kit [24]. However, in many districts, the CHF revenue which can be used to finance drug and supply purchases or the repair of equipment, minor facility renovation is only partially used. Such funds are often held at districts accounts, and facilities have to make claims for these resources. However, in many cases the facilities do not claim due to a lack of provider understanding of the process of fund allocation which is often not transparent [25].

Concerns about the lack of inclusion of referral care and associated transport costs in the benefit package have also been reported elsewhere [15]. The qualitative data in our study highlighted that respondents would find insurance more attractive if it covered higher cost referral services rather than primary care which is generally inexpensive due to the exemption policy for many services. However, this needs to be weighed against the potential impact on premiums of the inclusion of such services. Recent moves in Tanzania to promote engagement with the private sector in the provision of quality health services in areas with limited public facilities through service agreements (or contracts) [24] should facilitate a widening of the benefit package. A number of districts have entered into contract with faith-based hospitals to provide referral care to CHF members, which has been associated with higher enrolment rates [23]. It is also expected the primary health service development program (often referred as Mpango wa Maendeleo ya Afya ya Msingi (MMAM)) will improve service accessibility and availability by increasing the number of skilled staff, drugs and other medical supplies to the facilities.

Two low income countries in Sub-Saharan Africa have achieved high health insurance coverage: Rwanda and Ghana. In Rwanda, coverage is over 80% and in Ghana over 60% coverage has been attained [11,13,14]. In Rwanda, insurance enrolment is incentivised through a performance based financing scheme, which also aims to improve health care services [11,12]. In Ghana the scheme includes both formal and informal sector workers [13,14]. Tanzania recently piloted a pay for performance (P4P) scheme which it plans to roll out nationally [42]. Including CHF coverage as one of the P4P targets might offer an effective mechanism of increasing coverage, especially if parallel improvements in service delivery are noted. Further gains in terms of scheme design and revenue availability could be made by formerly linking the CHF to the NHIF, a mandatory scheme for the formal sector [25]. The takeover of the management of CHF by the NHIF is a positive step towards the ultimate merger of these schemes [25]. However, at present CHF administration costs remain substantial raising concerns about financial sustainability [43].

This study suffers from a number of limitations. We were unable to quantitatively assess the determinants of drop out, as there were insufficient numbers of drop outs within our uninsured sample. The analysis focused on community level stakeholders, so we did not examine the views of higher level stakeholders that may have shed more light on institutional factors constraining enrolment.

## Conclusion

There is a combination of demand and supply side factors constraining sustained enrolment in the CHF among the population. CBHI design needs to recognise these factors and build in mechanisms to attract members whilst deterring from adverse selection, stimulating the supply side to meet client expectations whilst ensuring financial viability. Raising awareness and understanding of the risk pooling principle is also essential among the population. Scheme managers need to ensure that the scheme remains appealing to the informal sector as a whole and not just the poor. Ideally, CBHI needs to be introduced alongside other initiatives to ensure effective use of funds generated to enable service quality improvements to members. Referral services should ideally be prioritised for inclusion in the benefit package and appropriate contractual relations are required to facilitate this process. Given the challenges of appropriate design, and the costs of administering the CHF, integration with existing national health insurance schemes should be considered. Tax funding is likely to remain the only viable mechanism for funding the poorest.

### Endnotes

^a^The exchange rate at the time of the study 2008 was Tanzanian shilling (Tsh) 1178 to US$1.

^b^National Health Insurance Funds (NHIF) is a compulsory health insurance covering civil servants officially introduced in 2001.

## Abbreviations

CBHI: Community Based Health Insurance
CHF: Community Health Fund
CHSB: Council Health Service Board
DC: District council
FGD: Focus Group Discussion
HFGC: Health Facility Governing Committee
OOP: Out of pocket

## References

[1] Preker AS, Carrin G, Dror D, Jakab M, Hsiao W, Arhin-Tenkorang D (2002). Effectiveness of community health financing in meeting the cost of illness. Bull World Health Organ.

[2] Jütting JP (2004). Do community-based health insurance schemes improve poor people’s access to health care? Evidence from rural Senegal. World Dev.

[3] Ekman B (2004). Community-based health insurance in low-income countries: a systematic review of the evidence. Health Policy Plan.

[4] Kamuzora P, Gilson L (2007). Factors influencing implementation of the Community Health Fund in Tanzania. Health Policy Plan.

[5] De Allegri M, Kouyaté B, Becher H, Gbangou A, Pokhrel S, Sanon M, Sauerborn R (2006). Understanding enrolment in community health insurance in sub-Saharan Africa: a population-based case–control study in rural Burkina Faso. Bull World Health Organ.

[6] Ron A (1999). NGOs in community health insurance schemes: examples from Guatemala and the Philippines. Soc Sci Med.

[7] Bennett S, Creese A, Monasch R (1998). Health Insurance Schemes for People Outside Formal Sector Employment. WHO/ARA/CC/98.1.

[8] Ranson KM, Sinha T, Chatterjee M, Acharya A, Bhavsar A, Morris SS, Mills AJ (2006). Making health insurance work for the poor: learning from the Self-Employed Women's Assocoation's (SEWA) community-based health insurance scheme in India. Soc Sci Med.

[9] Acharya A, Vellakkal S, Masset E, Satija A, Burke M, Ebrahim S (2012). Systematic Review Impact of National Health Insurance for the Poor and the Informal Sector in Low- and Middle-Income Countries by.

[10] Spaan E, Mathijssen J, Tromp N, McBain F, Have AT, Baltussen R (2012). The impact of health insurance in Africa and Asia: a systematic review. Bull World Health Organ.

[11] Twahirwa A (2008). Sharing the burden of sickness: mutual health insurance in Rwanda. Bull World Health Organ.

[12] Lu C, Chin B, Lewandowski JL, Basinga P, Hirschhorn LR, Hill K, Murray M, Binagwaho A: **Towards universal health coverage: an evaluation of Rwanda Mutuelles in its first eight years.** *PLoS One* 2012, **7**(6). 10.1371/journal.pone.0039282 PMC3377670 22723985

[13] Mills A, Ataguba JE, Akazili J, Borghi J, Garshong B, Makawia S, Mtei G, Harris B, Macha J, Meheus F, McIntyre D (2012). Equity in financing and use of health care in Ghana, South Africa, and Tanzania: implications for paths to universal coverage. Lancet.

[14] Amporfu E: **Equity of the premium of the Ghanaian national health insurance scheme and the implications for achieving universal coverage.** *Int J Equity Health* 2013, **12**(4). 10.1186/1475-9276-12-4 PMC3585509 23294982

[15] Carrin G, Waelkens M-P, Criel B (2005). Community-based health insurance in developing countries: a study of its contribution to the performance of health financing systems. Trop Med Int Health.

[16] Okello F, Feeley F (2004). Socioeconomic Characteristics of Enrollees in Community Health Insurance Schemes in Africa.

[17] Jehu-Appiah C, Aryeetey G, Spaan E, de Hoop T, Agyepong I, Baltussen R (2011). Equity aspects of the national health insurance scheme in Ghana- who is enrolling, who is not and why?. Soc Sci Med.

[18] Criel B, Waelkens M-P (2003). Declining subscription to maliando mutual health organization in Guinea-Conakry (West Africa): what is going wrong?. Soc Sci Med.

[19] Bonu S (2003). Using willingness to pay to investigate regressiveness of user fees in health facilities in Tanzania. Health Policy Plan.

[20] Ozawa S, Walker DG (2009). Trust in the context of community-based health insurance schemes in Cambodia: villagers’ trust in health insurers. Adv Health Econ Health Serv Res.

[21] Alkenbrack S, Jacobs B, Lindelow M (2013). Achieving universal health coverage through voluntary insurance: what can we learn from the experience of Lao PDR?. BMC Health Serv Res.

[22] De Allegri M, Sanon M, Sauerborn R (2006). “To enrol or not to enrol?”: A qualitative investigation of demand for health insurance in rural West Africa. Soc Sci Med.

[23] Macha J, Harris B, Garshong B, Ataguba JE, Akazili J, Kuwawenaruwa A, Borghi J (2012). Factors influencing the burden of health care financing and the distribution of health care benefits in Ghana, Tanzania and South Africa. Health Policy Plan.

[24] Stoermer M, Hanlon P, Tawa M, Macha J, Mosha D (2012). Community Health Funds (CHFs) in Tanzania: Innovations Study.

[25] Borghi J, Maluka S, Kuwawenaruwa A, Makawia S, Tantau J, Mtei G, Ally M, Macha J: **Promoting universal financial protection: a case study of new management of community health insurance in Tanzania.** *Health Res Policy Syst* 2013, **11**(21). 10.1186/1478-4505-11-21 PMC3686629 23763711

[26] McIntyre D (2008). Beyond fragmentation and towards universal coverage: insights from Ghana, South Africa and the United Republic of Tanzania. Bull World Health Organ.

[27] Mtei G, Mulligan J (2007a). Community Health Funds in Tanzania: A literature review.

[28] Maluka SO (2013). Why are pro-poor exemption policies in Tanzania better implemented in some districts than in others?. Int J Equity Health.

[29] URT (2001). The Community Health Fund Act, 2001. Acts Supplement to the Gazette of the United Republic of Tanzania, No. 14 Vol 82.

[30] Humba E (2011). Pioneering social health insurance in Tanzania: The case of the National Health Insurance Fund (NHIF). Improving Access through Effective Health Financing.

[31] Dong H, De Allegri M, Gnawali D, Souares A, Sauerborn R (2009). Drop-out analysis of community-based health insurance membership at Nouna, Burkina Faso. Health Policy.

[32] Dong H, Kouyate B, Cairns J, Mugisha F, Sauerborn R (2003). Willingness-to-pay for community-based insurance in Burkina Faso. Health Econ.

[33] Witter S (2009). Service- and population-based exemptions: are these the way forward for equity and efficiency in health financing in low-income countries?. Adv Health Econ Health Serv Res.

[34] Zhang L, Wang H (2008). Dynamic process of adverse selection: evidence from a subsized community- based health insurance in reual China. Soc Sci Med.

[35] Thornton RL, Hatt LE, Field EM, Islam M, Diaz FS, Gonzalez MA (2010). Social security health insurance for the informal sector in Nicaragua: a randomized evaluation. Health Econ.

[36] Gnawali DP, Pokhrel S, Sie A, Sanon M, De Allegri M, Souares A, Dong H, Sauerborn R (2009). The effect of community-based health insurance on the utilization of modern health care services: evidence from Burkina Faso. Health Policy.

[37] Kyomugisha EL, Buregyeya E, Ekirapa E, Mugisha JF, Bazeyo W (2009). Strategies for sustainability and equity of prepayment health schemes in Uganda. Afr Health Sci.

[38] Kiwara A (2007). Group premiums in micro health insurance: experiences from Tanzania. East Afr J Public Health.

[39] Parmar D, Souares A, de Allegri M, Savadogo G, Sauerborn R (2012). Adverse selection in a community-based health insurance scheme in rural Africa- Implications for introducing targeted subsidies. BMC Health Serv Res.

[40] Cofie P, De Allegri M, Kouyate B, Sauerborn R (2013). Effects of information, education, and communication campaign on a community-based health insurance scheme in Burkina Faso. Glob Health Action.

[41] Basaza RK, Criel B, Van der Stuyft P (2010). Community health insurance amidst abolition of user fees in Uganda: the view from policy makers and health service managers. BMC Health Serv Res.

[42] Borghi J, Mayumana I, Mashasi I, Binyaruka P, Patouillard E, Njau I, Maestad O, Abdulla S, Mamdani M (2013). Protocol for the evaluation of a pay for performance programme in Pwani region in Tanzania: a controlled before and after study. Implement Sci.

[43] Borghi J, Makawia S, Kuwawenaruwa A: **The administrative costs of community-based health insurance: a case study of the community health fund in Tanzania.** *Health Policy Plan* 2013, 1–9. doi:10.1093/heapol/czt093. 10.1093/heapol/czt093 PMC4287190 24334331

